# Utility of Cone-Beam CT for Bronchial Artery Embolization and Chemoinfusion: A Single-Institution Retrospective Case Series

**DOI:** 10.1007/s00270-022-03148-5

**Published:** 2022-04-19

**Authors:** Michael Y. Liu, Steven C. Rose, Alexander Loh, Michael Taddonio, Jonas W. Redmond, Quinn C. Meisinger, Jeet Minocha, Zachary T. Berman

**Affiliations:** 1 Department of Radiology, University of California San Diego Medical Center, 200 West Arbor Drive, San Diego, CA 92103 USA; 2 San Diego School of Medicine, University of California, San Diego, CA USA

**Keywords:** Embolization, Bronchial artery, Cone-beam CT

## Abstract

**Purpose:**

To describe the technique and document utility of adjunctive cone-beam CT (CBCT) in patients undergoing bronchial artery embolization (BAE) or chemoinfusion (BAC).

**Materials and Methods:**

Between August 2010 and February 2021, 26 patients (62 bronchial arteries) were evaluated with CBCT in addition to the usual digital subtraction angiography (DSA) during BAE or BAC. 19 patients (43 arteries) underwent BAE for hemoptysis; 7 patients (19 arteries) had BAC for palliation of lung malignancy. Retrospective review of procedural reports and the archived DSA and CBCT images was assessed for (1) whether CBCT findings added unique diagnostic information prior to treatment of target arteries compared to DSA alone; and (2) whether these unique CBCT findings led to modification of embolization or chemoinfusion technique.

**Results:**

In 61 of 62 (98%) interrogated bronchial arteries, CBCT provided additional unique diagnostic information over planar DSA, primarily cross-sectional assessment of the spinal canal for spinal arteries. In 46/62 (74%) of the bronchial arteries the unique information did not lead to a change in therapeutic technique. In 15 bronchial arteries (24%), the added information from CBCT led to change in embolization and/or chemoinfusion technique. Embolization of one small unrecognized spinal artery branch (1.6%), which was missed intra-procedurally but retrospectively seen on CBCT led to transient spinal cord ischemia.

**Conclusions:**

These results suggest that adjunctive use of CBCT technique may improve diagnostic confidence from information provided by DSA in nearly all cases of BAE and BAC leading to improved therapeutic targeting or change in technique of embolization or chemoinfusion.

## Introduction

Bronchial artery (BA) interventions, most notably bronchial artery embolization (BAE), have been well established for treatment of hemoptysis with high success rates [1]. Less commonly bronchial artery chemoinfusion (BAC) has been used for cytotoxic palliation of lung malignancies [2]. Navigating the BA anatomy for treatment can be challenging given the variable number, ectopic origins, and anatomical course of the bronchial arteries, as well as the shared circulations with intercostal, spinal, and chest wall arteries [3]. It is important to thoroughly define the arterial anatomy prior to embolization/infusion to ensure targeted treatment to the pathology while preventing non-target embolization. The most serious complications from non-target embolization include spinal cord ischemia/infarction reported to occur in 0.6–4.4% of patients [1].

Cone-beam CT (CBCT) has been demonstrated in other transcatheter therapies to be a useful adjunct to DSA (4). The purpose of this study was to describe the technique and evaluate the usefulness of CBCT technique during BA interventions by determining 1) whether CBCT increased operator confidence (i.e., provided relevant diagnostic information not visualized by DSA) of targeting compared to DSA alone and 2) whether CBCT provided information not available on DSA that led to modification of embolization or chemoinfusion technique.

## Materials and Methods

This study was a retrospective institutional review of archived images and procedural reports in all patients who underwent BAE or BAC of selectively catheterized bronchial arteries that were potential candidates for embolization or chemoinfusion in which CBCT was used. These bronchial arteries were defined as systemic arteries that directly supply lung parenchyma arising either from the aorta or intercostal arteries.

### Study Population

The institutional review board (IRB) approved retrospective review of cases between August 2010 and February 2021. There were a total of 26 patients (12 males and 14 females; mean age 55 years) with 62 total DSA image series from arteries suspected to supply targeted pathology which underwent bronchial artery interventions with the adjunctive use of CBCT. Of these 26 study patients, 19 underwent BAE and 7 underwent BAC. The indications for bronchial arteriography, intervention, and underlying pathologies are summarized in Table 1.

**Table 1:** Indications for bronchial artery intervention

Indication for bronchial artery intervention and underlying pathology	
*Chemoinfusion (N* = *7 Patients)*	
Squamous cell carcinoma	3
Non-small cell carcinoma	1
Nerve sheath sarcoma	2
Mesothelioma	1
*Hemoptysis (N* = *19 Patients)*	
Lung malignancy	4
Aspergillosis	5
Pulmonary tuberculosis	2
Pulmonary embolism	1
Pneumonia	4
Bronchial arteriovenous malformation	1
Behcet’s disease	1
Loeys–Dietz syndrome	1

### Angiographic and Cone-Beam CT Technique

All patients underwent preprocedural contrast enhanced chest computed tomography angiography (CTA) scans using thin slice acquisition (1.5 mm). Catheter aortography was not routinely performed during the embolization procedures since bronchial arteries were usually identified by prior CTA, which allowed the operators to proceed directly to catheterization of the bronchial or intercostal arteries. If the vessel looked to be a potential embolization or chemo-infusion candidate, CBCT was performed. The origins of target bronchial arteries were selectively catheterized using 5 Fr base catheters (usually Cobra [Cook Medical, Bloomington, IN], Mikaelson [Angiodynamics, Queensbury, NY], or Shetty shaped [Cook Medical, Bloomington, IN]), and confirmed with DSA. If an appropriate candidate artery for BAE or BAC was identified, more selective and stable bronchial artery catheterization was performed with 2.4–2.9 Fr microcatheters (most commonly Maestro, Merit Medical; Salt Lake City, UT or Progreat, Terumo; Somerset, NJ).

CBCT was used prior to embolization or chemoinfusion to address specific diagnostic questions, including: (1) Was the targeted pathology within the perfused vascular territory? (2) Did opacified arteries enter the spinal canal or perfuse other critical non-targeted structures? Or (3) Were portions of the targeted pathology not opacified and therefore stimulated a search for additional target arteries to be treated?

CBCT was performed injecting nonionic contrast material (Visipaque 320, GE Healthcare; Cork, Ireland) diluted 1:1 with normal saline. Image acquisition was achieved using cone-beam CT software (Artis, Siemens Inc; Munich, Germany). Contrast material was power injected at a rate of 0.3–1.0 mls/sec, based on the subjective assessment of bronchial arterial diameter and blood flow velocity as determined on an antecedent DSA. To obtain both an arterial phase and parenchymal phase contrast enhancement, contrast material was injected for 20 s. Image acquisition was initiated 12–15 s after contrast material injection was started; images were acquired over 5–6 s with image intensifier rotation of 220 degrees. Patients were positioned supine on the angiographic table with both arms abducted fully when feasible. In all cases, the region of interest for CBCT acquisition included the bronchial artery and thoracic spinal canal. Images were presented on an independent 3D workstation (Leonardo Syngo X, Siemens Inc; Munich, Germany) as three simultaneous orthogonal CT images as well as a volume rendered image. The operating physician then analyzed the axial, sagittal, and coronal CT images, with attention to bronchial arterial anatomy, presence of arterial branches to non-targeted structures (e.g., spinal canal), and the degree and location of parenchymal opacification. The volume rendered image data set was optimized by operator-dependent exclusion of extraneous anatomy and adjustment of windowing levels to depict the vascular anatomy as a three-dimensional vascular cast. The operators’ estimates of time required for CBCT setup was approximately 60 s, image acquisition 20 s, image manipulation and analysis 2–5 min, for an estimate time investment of approximately 4–7 min per CBCT image set. The volume of contrast injected and radiation exposure specific to CBCT acquisition were not recorded.

### DSA Injection Technique

The contrast material injection rate was subjectively estimated based on fluoroscopic hand injections. The rate selected varied based on vessel size and tissue vascularity ranging from 0.3 ml per sec (the lowest value achievable on the power injector) to 1.0 ml per sec. DSA image series were considered adequate if contrast refluxed to the vessel origin or into the aorta. The injection duration was set at 10 s with contrast volume tenfold the injection rate. All patients were coached to remain motionless and suspend respiration in full inspiration for the duration of image acquisition, for both DSA and CBCT.

### Embolization Technique

After the candidate vessel(s) were evaluated with both DSA and CBCT and deemed suitable for intervention, the operators commonly performed embolization with calibrated microspheres (Embozene microspheres, Boston Scientific, Natick, Massachusetts) (*n* = 11). Depending on the operator and characteristics of the targeted vessel, polyvinyl alcohol [Contour PVA embolization Particles; Boston Scientific] (*n* = 3), n-butyl cyanoacrylate adhesive liquid (n-BCA) [Histoacryl; B. Braun, Melsungen, Germany] (*n* = 5), ethylene–vinyl alcohol copolymer (Onyx) [ ev3, Irvine, CA] (*n* = 2), and coils [Detach-18, Cook Medical Inc] (*n* = 2), a mixture of Embozene with n-BCA (*n* = 8) or PVA and n-BCA (*n* = 1) were also used. In all cases, embolization end points included patent bronchial artery origins, with the absence of opacification of targeted pathology and reduction in number and degree of branch arteries.

### Chemoinfusion Technique

The BAC technique has been previously described in the literature [2]. Briefly, BAC was performed using transcatheter intra-arterial infusion of supratherapeutic liquid cisplatin (150 mg/m^2^) to treat various lung malignancies, with simultaneous IV infusion of thiosulfate (9 g/m^2^) to bind free systemic cisplatin, a potent neurotoxin. Given the neurotoxicity of cisplatin, CBCT was routinely performed prior to each infusion to ensure that no candidate arteries for infusion provided angiographically occult supply to the spinal canal. In addition, the extent of tumor perfusion was assessed.

### Data Analysis

The patients’ imaging files were stored on the institutional PACS system and retrospectively reviewed by two board-certified Interventional Radiologists with experience in bronchial arterial interventions in conjunction with their procedure reports. Each report detailed the DSA and CBCT findings, including any intraprocedural changes as a result of the findings. The review occurred in chronological order of their acquisition: (a) pre-embolization DSA and (b) pre-embolization CBCT. No analysis was performed on post-embolization DSA, and no CBCT was performed after embolization. Divergent opinions were infrequent and resolved by mutual review and consensus opinion. Blinded review was impractical since clinical context and the anatomic context (provided by the pre-procedure CTA) were imperative to understand the angiographic and CBCT findings.

Based on the procedure reports and independent review of the saved DSA and CBCT images, the findings of each artery’s CBCT were compared to the matching antecedent DSA and judged to fall into one of four categories: (1) DSA provided diagnostic information not available on CBCT; (2) CBCT added no additional information over DSA; (3) CBCT findings added unique diagnostic information relevant to therapeutic targeting without leading to a change in procedural management; (4) CBCT added unique diagnostic information that resulted in a change in intraprocedural management. The listed criteria for categories 3 and 4 are summarized in Table 2.

**Table 2:** Interpretive categories and criteria for placement regarding clinical utility of CBCT vis-a-vis antecedent DSA

Category 3 (Beneficial findings with increased diagnostic confidence without change in management)	Category 4 (Beneficial findings with increased diagnostic confidence and change in management)
Confirmed corresponding vascular supply with soft tissue opacification	Resulted in more distal catheter positioning to avoid non-target embolization
Clarified questionable or variant anatomy on DSA	Resulted in finding an alternative vessel source after contrast staining of normal tissue
Established correlation between vascular supply and tumor volume	Change in vessel catheterization to avoid spinal artery embolization
Confirmed the absence of spinal artery opacification	Unexpected protective coil embolization of vessels to prevent non-target embolization or to redirect blood flow
Identified angiographically occult tumors	Embolization of additional arteries to cover entire tumor volume

For patients who received embolization or chemoinfusion of more than one artery, each vessel was interpreted separately and was adjudicated to fall into categories 1–4 based on the corresponding CBCT findings. If a patient had multiple repeat interventions, each subsequent vessel interrogated with DSA and CBCT was added to the analysis. The frequency and percentage of each category were calculated.

## Results

In this study, a total of 62 arteries in 26 patients were suspected to supply the source of hemoptysis or lung malignancy based on DSA. Each artery was then interrogated with CBCT. Of the 26 patients, six patients underwent subsequent bronchial artery re-interventions (three for BAE for repeat hemoptysis and three for planned staged BAC of tumors).

**Fig. 1:**
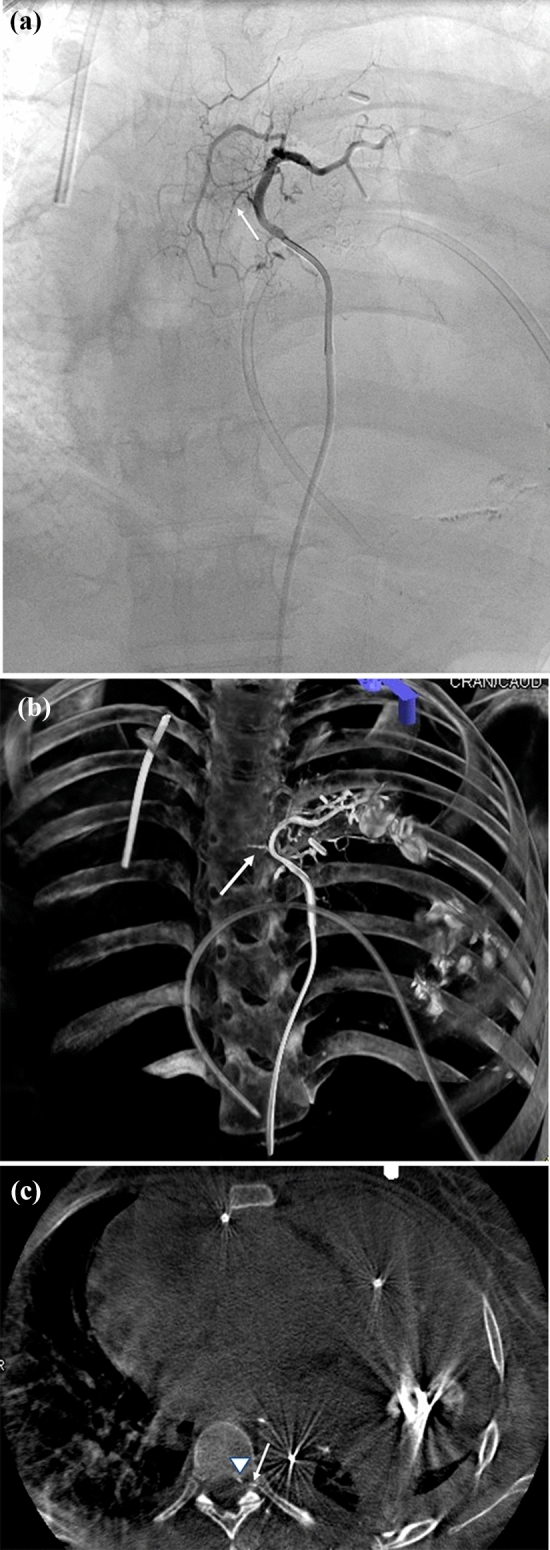
23-y/o female patient with history of hemoptysis from a left thorax synovial cell sarcoma. **a** DSA coronal image with selective injection of the left bronchial artery with visualization of the minute spinal artery branch seen only in retrospect (white arrow). **b**, **c** Volume-rendered image and axial CBCT demonstrating the small spinal artery branch—traversing the intervertebral foramen (white arrow) into the spinal canal (arrowhead)—which was overlooked at time of procedure

In no cases did DSA provide unique diagnostic information not available on CBCT (category 1) or that CBCT failed to provide unique additional information compared to DSA (category 2). In 46/62 (74%) of interrogated arteries for intervention, CBCT added unique diagnostic information relative to targeting of BAE or BAC therapy but without change in interventional technique. Specific findings included abnormal lung parenchyma staining and the absence of spinal artery opacification both features which were poorly assessed by DSA (Table 3, category 3). One case (1.6%) was prospectively a diagnostic miss and resulted in spinal cord ischemia after embolization. Retrospective scrutiny detected a small spinal cord artery branch seen on the CBCT but not on retrospective analysis of the DSA in isolation (Fig. 1).

**Table 3:** Category 3 (**46 vessels**) features of cases in which CBCT added information not available on DSA alone and did not result in modification of BAE or BAC technique

CBCT imaging benefit	Bronchial artery embolization (*n* = 43)	Chemoinfusion (n = 19)
Confirmed targeted vascular supply/abnormal lung parenchymal staining and identified the absence of spinal canal opacification	31	15

**Fig. 2.**
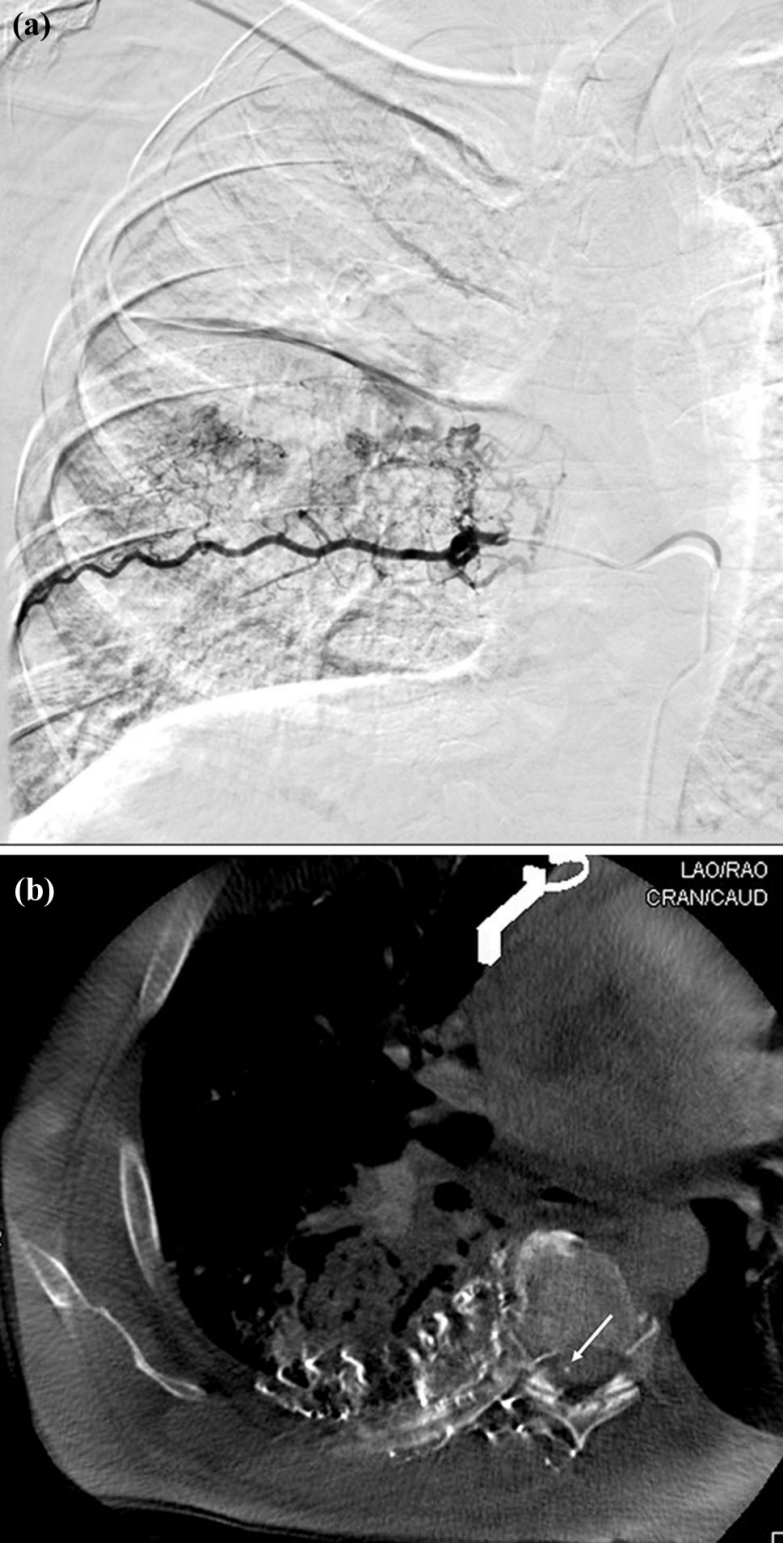
62-y/o male with history of tuberculosis and aspergilloma, who presented with hemoptysis. **a** Coronal DSA interrogation of the right T7 intercostal artery demonstrates abnormal lung opacification without clear evidence of a spinal artery. **b** CBCT at this region demonstrating arterial perfusion to the anterior spinal artery (white arrow), which was occult on DSA. This prompted disengagement of the corresponding vessel and prevented a potential non-target embolization

In 15/62 (24%) of interrogated vessels, the added information from CBCT *did* lead to a modification of interventional technique (Table 4; category 2). In 5/15 arteries, CBCT prospectively identified spinal arteries not initially visualized on DSA that led to repositioning of the microcatheter prior to embolization (Fig. 2). In 8/15 interrogated arteries, DSA tissue opacification was suspected to involve targeted pathological lung parenchyma, but CBCT demonstrated that the tissue opacification was normal lung parenchyma or chest wall, which led to a search for an alternative arterial supply. In 2/15 interrogated arteries, CBCT documented unique diagnostic findings that revealed additional embolization targets.

**Table 4:** Category 4 (**15 vessels**) features of cases in which CBCT added information not available on DSA alone but did result in modification of BAE or BAC technique

CBCT imaging benefit		Bronchial artery embolization (*n* = 43)	Chemoinfusion (*n* = 19)
Microcatheter repositioning	To avoid a DSA occult spinal artery	4	1
	To avoid unnecessary embolization	6	2
Identified additional vessels for embolization		1	1

### Complications

Non-target particle embolization of a small, prospectively unrecognized spinal artery branch (1.6%) resulted in transient spinal cord ischemia, which resolved within a week of the patient’s hospital stay. Follow-up MRI thoracic spine demonstrated no evidence of acute infarct. No other complications were present.

## Discussion

For BA interventions, DSA remains the standard intraprocedural imaging technique. However, it is limited by vessel superimposition, motion artifact, and limited range for display of radiation attenuation. Alternatively, CBCT has intrinsic features that can be used to overcome DSA limitations. It allows interactive interrogation to identify the three-dimensional (3D) relationship of blood vessels, lung parenchyma, soft tissue, and osseous structures. The range of radiation attenuation display is greater, and volume rendered images can better present the nature of these complex arteries. CBCT does come with a cost; it increases the volume of contrast injected, radiation exposure to the patient, and procedural time and effort.

CBCT has been extensively studied in extrapulmonary locations, especially to guide transcatheter therapies for treatment of liver malignancies [4–11]. Pung and colleagues have demonstrated improved sensitivity for detection of tumors (90% vs. 67%) and tumor feeding arteries (93% vs. 55%) with CBCT compared to DSA alone [4]. Multiple investigators have also reported that CBCT provided unique information that resulted in modification of 28 to 81% of TACE procedures [5–8]. Potential survival benefit related to adjunctive CBCT with TACE has also been reported [9–11].

Prior reports on use of CBCT for BA interventions are few. Grosse and colleagues compared preprocedural chest CT and flush aortic DSA to flush aortic CBCT (not a technique used in the current report) in 17 consecutive patients undergoing BAE for treatment of hemoptysis [12]. With respect to identification of the number and origins of bronchial arteries, diagnostic confidence, and image quality, CBCT was superior to both pre-procedural CT (*p* = 0.03) and flush DSA aortography (*p* = 0.001). He and associates used selective CBCT in 26 arteries thought likely to supply the pathologic entity causing hemoptysis [13]. CBCT determined whether the targeted artery supplied the source of bleeding in 24 arteries (92%). In 16/24 (66.7%) arteries, CBCT demonstrated that the targeted artery did not supply the presumed source of bleeding and thus avoided unnecessary embolization. In 8/24 (33.3%), CBCT confirmed that the targeted artery did supply the presumed source of bleeding, which led to BAE.

The results regarding CBCT’s impact in modification of interventional technique in the presented series of 62 interrogated arteries (26 patients) are similar to findings for other interventional procedures, such as TACE [5–8]. By intent, the field of view in all instances of CBCT was designed to include the thoracic spine. In 46/62 (74%) of interrogated arteries using CBCT, the added information increased operator diagnostic confidence without changing the interventional technique by confirming DSA did not over-look any spinal arteries and that the targeted artery supplied the source of pathology. In these cases that confirmed coverage of targeted pathology, the confirmation removed potential uncertainty present based on DSA alone. In multiple cases, the perfused territory was not the targeted pathology suspected on DSA but represented either posterior chest wall or paraspinous musculature seen on CBCT.

The case of transient weakness and paresthesia following non-target embolization of an unrecognized small spinal artery branch bears scrutiny. This unrecognized spinal artery branch did not demonstrate the classic “hairpin” appearance on DSA. Retrospective identification of the spinal artery only occurred after CBCT identified the spinal artery on cross-sectional axial CT type presentation, which then provided a volume rendered image of the same vessel to match the DSA projection. This represented a 1.6% diagnostic error rate.

In 15/62 (24%) of interrogated vessels, the use of CBCT led to alteration of interventional technique. The most important aspect relates to potential safety. In five arteries, the anterior spinal artery was identified prospectively by CBCT and not visualized on DSA (Fig. 2); the microcatheters were either removed and no embolization was performed or advanced further to avoid the spinal artery branch. The one additional spinal artery identified retrospectively on CBCT but not by DSA alone would have increased the total of spinal arteries identifiable on CBCT that did or should have modified embolization technique to six. This prospectively missed spinal artery on CBCT underscores the importance of careful scrutiny of cross-sectional images of the spinal canal. The other important feature relates to improved therapeutic targeting, ideally to treat completely but only the pathologic tissue. In 8 interrogated arteries, CBCT identified arteries thought to supply targeted pathology based on DSA findings, but in fact supplied either normal lung parenchyma or chest wall, so BAE or BAC was withheld. In one other case, CBCT identified additional vascular supply to the targeted pathology, which led to embolization. In the remaining case, CBCT identified a suspected intercostal artery not supplying the tumor but was continuous with other intercostal arteries supplying the tumor. This led to protective coil embolization at the point of collateral circulation to prevent non-target chemoinfusion.

This study has several important limitations. A major limitation being that this study was not designed to address whether the adjunctive use of CBCT affected clinical efficacy or resulted in improved patient safety. Additionally, this study did not have a control arm, so a meaningful comparison between adjunctive use of CBCT and DSA alone could not be assessed. As a retrospective study, this study is subject to inherent selection or information bias. The results were limited to a small sample size from a single intuition and primarily one experienced operator; thus, the generalizability of results is unknown. This study was based on the dictated reports and stored images from the operating physicians and therefore dependent on the adequacy of what data were designated to be stored. Because of the retrospective nature, determining the specific questions in the operator’s mind to be answered by CBCT cannot be known with certainty. The impact of CBCT on injected volume of contrast material or patient radiation exposure was not recorded.

In conclusion, because CBCT provided multiplanar assessment of the spinal canal and the targeted pathologic parenchyma, it improved diagnostic confidence from the information provided by DSA in nearly all cases of BA interventions. The added unique information of CBCT, including visualization of arteries entering the spinal canal; determination of exact anatomic compartments of perfused tissue; clarification of totality of perfusion of targeted pathology; and identification of obscured structures by DSA misregistration led to modification of interventional technique for improved potential safety or targeting in 24% of the arteries included in this study. However, despite the use of CBCT, there was one case of non-target embolization of a spinal artery branch, which was only seen retrospectively with the aid of CBCT. The authors hope that this serves as a cautionary tale that despite the improved visualization of spinal arteries with CBCT, it is still necessary to maintain high diagnostic vigilance with this and any other imaging techniques used.
